# Development of a Novel Robotic Rehabilitation System With Muscle-to-Muscle Interface

**DOI:** 10.3389/fnbot.2020.00003

**Published:** 2020-02-18

**Authors:** Jae Hwan Bong, Suhun Jung, Namji Park, Seung-Jong Kim, Shinsuk Park

**Affiliations:** ^1^Department of Mechanical Engineering, Korea University, Seoul, South Korea; ^2^School of Dentistry, Seoul National University, Seoul, South Korea; ^3^College of Medicine, Korea University, Seoul, South Korea

**Keywords:** wearable robot, human-human interface, electromyogram, functional electrical stimulation, lower limb rehabilitation

## Abstract

In this study, we developed a novel robotic system with a muscle-to-muscle interface to enhance rehabilitation of post-stroke patients. The developed robotic rehabilitation system was designed to provide patients with stage appropriate physical rehabilitation exercise and muscular stimulation. Unlike the position-based control of conventional bimanual robotic therapies, the developed system stimulates the activities of the target muscles, as well as the joint movements of the paretic limb. The robot-assisted motion and the electrical stimulation on the muscles of the paretic side are controlled by on-line comparison of the motion and the muscle activities between the paretic and unaffected sides. With the developed system, the rehabilitation exercise can be customized and modulated depending on the patient’s stage of motor recovery after stroke. The system can be operated in three different modes allowing both passive and active exercises. The effectiveness of the developed system was verified with healthy human subjects, where the subjects were paired to serve as the unaffected side and the paretic side of a hemiplegic patient.

## Introduction

A large number of patients suffer from lower limb hemiplegia after experiencing a stroke. Post-stroke hemiplegic patients have impaired gait pattern and must undergo rehabilitation exercises to restore their normal gait pattern. Intensive rehabilitation exercises must be conducted within a golden period, between 3 and 6 months following the stroke within which most functional restoration takes place. However, rehabilitation processes often do not begin in a timely manner due to the limited number of therapists available to conduct the exercises. The process of rehabilitation requires significant time and effort for the therapists and therefore the number and duration of rehabilitation sessions hardly meet the demand. It is crucial that this problem be addressed as studies have indicated that increasing the amount of physiotherapy has a positive effect on functional recovery (Kwakkel et al., 2008; Huang and Krakauer, 2009; Kollen et al., 2009; Kwakkel, 2009; Marchal-Crespo and Reinkensmeyer, 2009).

In order to reduce the therapists’ workload and thus increase the patients’ accessibility of rehabilitation sessions, multiple kinds of robotic rehabilitation systems have been developed. However, some studies have raised concerns regarding robot-assisted rehabilitation systems due to the patient’s passivity in conducting the exercise (Israel et al., 2006; Hornby et al., 2008; Hidler et al., 2009). The degree of functional recovery during rehabilitation depends on the level of task difficulty and the amount of exercise actively conducted by the patient. In order to maximize the efficacy of rehabilitation exercises and thus functional recovery, patients must actively contract their appropriate muscles rather than passively depend on the robot to conduct the pre-programed motions (Hornby et al., 2008; Hidler et al., 2009). Studies have shown that low patient involvement and ease of exercise compromise the speed and outcome of functional recovery (Israel et al., 2006). Leaving the patient idle during the rehabilitation exercise risks wasting the golden period. In order to effectively make use of the golden period, patient involvement in the rehabilitation exercise must be maximized in a timely manner. Passive and active rehabilitation methods should be selected depending on the patient’s recovery phases. In the early stage of rehabilitation, the passive exercise is essential to provide the reference trajectories of the motion to patients in order to improve the movability and to reduce muscle atrophy (Jamwal et al., 2014). After recovering a certain degree of muscle strength, the active exercise is necessary to encourage voluntary muscle activation by the patient.

There are three types of control modes that are commonly used for robot-assisted rehabilitation: passive mode, active assist mode, and active resist mode (Marchal-Crespo and Reinkensmeyer, 2009; Pittaccio and Viscuso, 2011). In the passive mode, the patient solely depends on the robot movements that follow the reference trajectories generated by using a position-based trajectory tracking control method (Emken et al., 2008; Beyl et al., 2009; Vallery et al., 2009; Duschau-Wicke et al., 2010; Saglia et al., 2012; Hussain et al., 2013b; Jamwal et al., 2014). The reference trajectories are generated from the movements of the unaffected limb as in bimanual rehabilitation (Lum et al., 2004). Bimanual rehabilitation is a treatment method, in which the patient moves both paretic and unaffected limbs simultaneously. It has been reported that training both limbs in identical motion aids recovery by coupling symmetric proprioceptive feedback in both sides of the ipsilateral corticospinal pathway (Wolf et al., 1989; Burgar et al., 2000). In the active assist mode, the robot provides partial assistance to the patients who recovered muscle strength to produce a voluntary motion. The active resist mode is used to help strengthen the muscle forces of the patients by performing the exercise against a resistive force exerted by the robot (Poli et al., 2013). A number of rehabilitation robots employ the well-known impedance control strategy for the active assist mode and the active resist mode to encourage active participation of the patient and to adjust the dynamic relationship between robot position and contact force (Emken and Reinkensmeyer, 2005; Agrawal et al., 2007; Veneman et al., 2007; Wolbrecht et al., 2008; Roy et al., 2009; Duschau-Wicke et al., 2010; Hussain et al., 2013a; Koopman et al., 2013). In active operation modes with impedance control, it is difficult to stimulate and control the contraction of the specific target muscles that are necessary to generate the movement. In their meta-analyses for the effects of robot-assisted rehabilitation, Veerbeek et al. (2017) reported that robot-assisted rehabilitation can improve motor control ability and muscle strength in the paretic side, while the improvement does not appear significant. This issue may be alleviated with the aid of functional electrical stimulation (FES) that delivers low intensity electrical stimulation to a specific nerve or muscle to induce muscle contraction artificially. FES is known to be beneficial for improving motor ability and inducing changes in motor cortex excitability and functional cortical reorganization (Maffiuletti et al., 2011; Popović, 2014).

In this study, we aim to develop a robot-assisted rehabilitation system combined with the application of FES on the muscles in the lower limb to enhance the recovery process for hemiplegic stroke patients. Several studies have attempted to provide robotic rehabilitation therapies by using hybrid robotic rehabilitation systems (HRRS), where FES is applied in addition to volitional muscle contraction in order to induce further muscle contraction and thus muscle forces (Langzam et al., 2006a, b; Bulea et al., 2013; Chen et al., 2013). In those studies, the intensity of FES is controlled either by predefined stimulation pattern (Bulea et al., 2013) or by feedback control (Chen et al., 2013), which takes into account of the states of the paretic side only. The robotic system developed in this study employs electromyography (EMG) biofeedback signals from both unaffected and paretic sides to control the motion and muscle activities of the paretic limb. Unlike the position-based control of conventional bimanual robotic therapies, this feature aims to exploit the functional bimanual synergies at the level of muscle activities, as well as at the level of joint movements.

The HRRS developed in this study provides the patient with the passive and active exercises. During the passive exercise, a one-DOF rehabilitation orthosis for knee movement is controlled by a proportional-derivative (PD) controller to provide isokinetic exercise for the paretic leg. The desired position of the paretic leg is set to be the position of the unaffected leg, while the desired velocity is set constant. During the active exercise, both the orthosis motion and the FES intensity on the paretic side are controlled. The orthosis is controlled by admittance controller to generate a target interaction force between the orthosis and the paretic leg. FES applied to the paretic leg is modulated to generate appropriate muscle contraction to follow the knee joint motion of the unaffected leg. FES intensity is controlled by comparing the muscle activities of the paretic and unaffected legs. EMG is measured from Rectus Femoris (RF) – one of the knee extensor muscles. The measured EMG signals from both paretic and unaffected sides are processed and compared to modulate the FES intensity to induce the muscle activity for the RF on the paretic side close to that on the unaffected side. The passive and active operation modes can be selected depending on the patient’s stage of motor recovery after stroke. It was reported that the functional restoration of knee extensor muscles, such as RF, plays an important role in regulating comfort and gait speed of hemiplegic patients (Hsu et al., 2003).

This paper is organized as follows: section “Materials and Methods” describes the developed rehabilitation system. Section “Experiments and Results” explains the experimental setup and shows performance evaluation of the developed rehabilitation system. Section “Conclusion” summarizes the major points of the system performance and concludes the study.

## Materials and Methods

### System Overview

The HRRS developed in this study consists of four major components: an exoskeleton robot (ATO, KIST, Seoul, South Korea), sEMG sensors with wireless transmission devices (Trigno Lab, Delsys Inc., United States), a FES device (Rehastim, Hasomed GmbH, Germany), and a self-designed knee angle measurement device.

**FIGURE 1:**
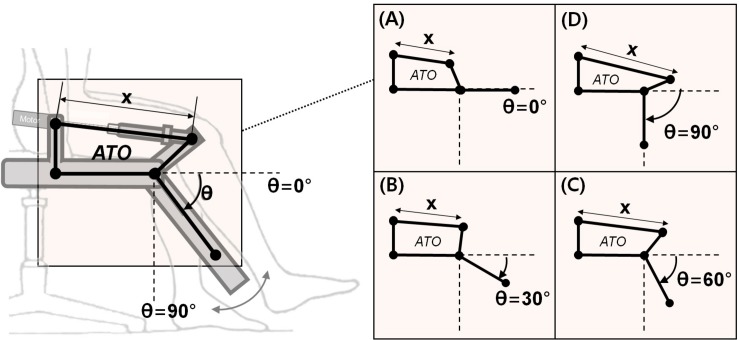
The joint angle (θ) configuration of ATO: **(A)** θ = 0° when ATO is in the position of full knee extension, **(B–D)** increasing of θ to the 90° while ATO operating in clockwise by changing the linear length of *x*.

**FIGURE 2:**
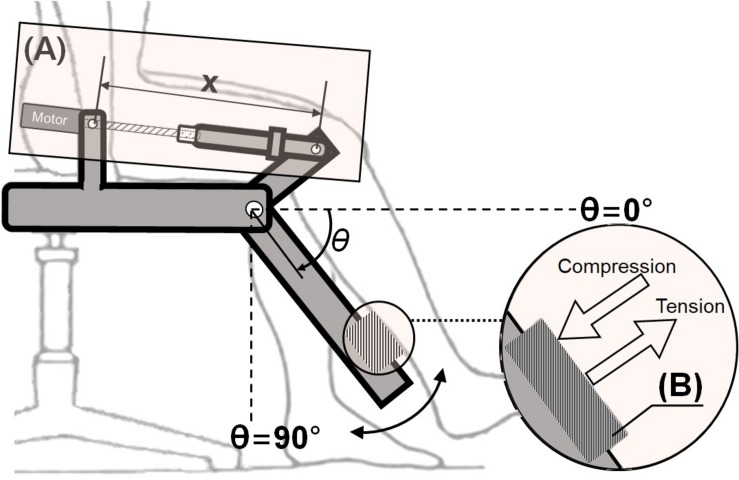
Actuator and sensor in ATO system: **(A)** Linear actuator with a ball screw and a BLDC motor, **(B)** load cell to measure interaction force between the paretic leg and ATO.

ATO system developed in the previous study (Lee et al., 2013) is an exoskeleton-type robotic orthosis for one-DOF sagittal knee motion (extension-flexion). The patient’s leg is attached to ATO system by a brace at the calf. As shown in Figure 1, the joint angle θ is defined 0° when the patient’s knee is fully extended along with ATO (Figure 1A). While ATO moves in the direction of knee flexion, the joint angle θ is increased as depicted from Figures 1A–D. For actuation of the joint angle θ ranging from 0° (Figure 1A) to 90° (Figure 1D), a linear actuator equipped with a ball screw and a BLDC motor (Maxon EC-4pole 200W) is implemented (see Figure 2A). The linear actuator changes the length *x* in Figure 2A, which results in joint angle (θ) regulation. The load cell equipped at the brace measures the interaction force between the patient’s leg and ATO (see Figure 2B). The patient’s leg is strapped tightly to the brace so that the load cell can measure both tensile and compressive interaction forces. The muscle force including volitional portion and FES induced portion is observed by the measured interaction force. For instance, observation of large tensile interaction force means the patient generates the large muscle force in the direction of knee extension. As another example, observation of compressive interaction force same as leg weight means the patient fully relies on ATO and does not generate any muscle force. The measured interaction force is also used to control the sagittal knee motion of ATO through admittance control (see section “Active Assist Mode” and section “Active Resist Mode”).

**FIGURE 3:**
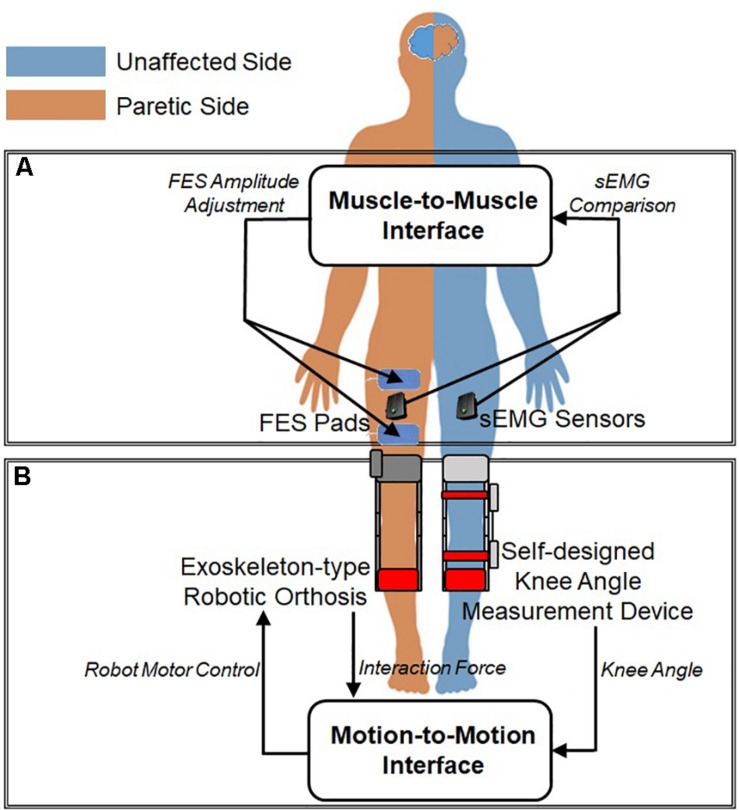
Interfaces between unaffected and paretic legs: **(A)** Muscle-to-Muscle Interface and **(B)** Motion-to-Motion Interface.

As illustrated in Figure 3, the developed system employs two types of interfaces between the unaffected and paretic sides of the patient: muscle-to-muscle Interface and motion-to-motion Interface. The muscle-to-muscle Interface, described in Figure 3A, modulates the amplitude of FES on the paretic leg based on the difference in the readings from sEMG sensors on the unaffected and paretic legs. The motion-to-motion Interface, described in Figure 3B, controls the sagittal knee motion of ATO and thus guides the knee joint motion of the paretic side based on the knee joint motion of the unaffected side and the interaction force between ATO and the paretic leg. The knee joint motion of the unaffected side was measured by the self-designed knee angle measurement device consisting of a hard-type commercial knee brace and a goniometer. The goniometer was attached on the side of hard-type commercial knee brace to measure the knee motion.

#### Muscle-to-Muscle Interface

The muscle-to-muscle interface compares the EMG data from the RF on the paretic and unaffected legs to adjust the amplitude of FES applied on the paretic leg. The EMG records electrical activity in a muscle, which reflects degree of muscle activation. The EMG data from the RF on the paretic and unaffected legs are measured and processed. Based on the difference between the two processed EMG readings, the intensity of FES is modulated and applied to the RF on the paretic leg. Through FES, the patients can train to learn their appropriate amount and timing of muscle activation of the RF on the paretic leg.

Since the raw EMG readings from different muscles show different characteristics in amplitude and frequency, it is difficult to compare the raw EMG signals without preprocessing (De Luca, 1997). Moreover, the EMG data from the paretic side contains stimulus artifacts induced by FES. In this study, the EMG signals are processed using filtering, feature extraction, and normalization techniques. The filter was designed using a combination of a blanking window and a comb filter. The blanking window is used for the EMG signal from the paretic side to nullify stimulation artifacts from the first 25 ms of the FES pulse (Frigo et al., 2000). After applying the blanking window, the comb filter removes harmonic artifacts and thus isolates the volitional component of the EMG signal (Frigo et al., 2000). The comb filter is a finite impulse response (FIR) filter and can be expressed as follows:

(1) $$y\,\left(t\right)=\frac{x\,\left(t\right)-x\,\left(t-T\right)}{\sqrt{2}}$$

In equation (1), *x(t)* and *y(t)* are the raw EMG and the filtered EMG at time *t*. *T* denotes a time period of FES.

Feature extraction technique is applied on the filtered EMG using waveform length, which is effective for extracting time-domain features including waveform amplitude, frequency, and duration (Phinyomark et al., 2010; Negi et al., 2016; Veer and Sharma, 2016). The waveform length can be expressed as follow:

(2) $$y=\sum_{i=0}^{N-1}\left|x_{i+1}-x_{i}\right|$$

In equation (2), *x* and *y* are the filtered EMG and the waveform length, respectively. *N* is a constant related to the number of samples to be used for calculating waveform length. In this paper, *N* was used for 160.

As a common normalization method, EMG signals are divided by a reference value. The reference value was taken by the maximum EMG value (Halaki and Ginn, 2012). Maximum activation for each subject was obtained beforehand while performing the task under maximum effort.

After the signal is processed according to procedures mentioned above, the difference between the processed EMG signals from RF on the paretic and unaffected legs is calculated. Based on the EMG difference, the amplitude of FES applied on the paretic leg is determined. If the EMG difference is less than 0.01, the FES amplitude is maintained. If the EMG difference is greater than 0.01 and the EMG signal from the RF on the unaffected leg is larger than that from the RF on the paretic leg, the FES amplitude is increased by 2 mA. If the EMG difference is greater than 0.01 and the EMG signal from the RF on the unaffected leg is smaller than that from the RF on the paretic leg, the FES amplitude is decreased by 2mA. Altogether, the FES amplitude is determined as follows:

(3) $$E\,M\,G=E\,M\,G_{u\,n\,a\,f\,f\,e\,c\,t\,e\,d}-E\,M\,G_{p\,a\,r\,e\,t\,i\,c}$$

(4) $$F\,E\,S_{i}=\left\{ \begin{array}{c} FES_{i-1}+2\;\left(EMG>0.01\right)\\ FES_{i-1}\;\left(|EMG|\leq 0.01\right)\\ FES_{i-1}-2\;\left(EMG<-0.01\right) \end{array}\right.$$

In equation (3), *EMG*_*unaffected*_ and *EMG*_*paretic*_ indicate the processed EMG signals from RF on the unaffected and paretic legs, respectively. In equation (4), *FES*_*i*_ is the amplitude of *i*^*th*^ FES applied to the RF on the paretic leg.

#### Motion-to-Motion Interface

The motion-to-motion interface maps the knee joint motion of the unaffected leg to the motion of joint movement of ATO worn on the paretic leg. The motion-to-motion interface can provide the patient with three types of control modes: passive mode, active assist mode, and active resist mode. In the passive mode, the interface conducts robotic motion assistance for the patient incapable of generating volitional muscle contractions. While the joint angle (θ) of ATO is controlled by the knee angle of the unaffected leg, the joint velocity of ATO is kept constant during extension and flexion. This type of isokinetic exercise has been reported to be appropriate in the early phase of rehabilitation (Cabri and Clarys, 1991). Two different active modes, the active assist mode and the active resist mode, are combined with the muscle-to-muscle interface described in section “Muscle-to-Muscle Interface.” The interface provides assistive or resistive forces to the paretic leg using admittance control based on the knee joint motion of the unaffected leg and the interaction force between the paretic leg and ATO.

**FIGURE 4:**
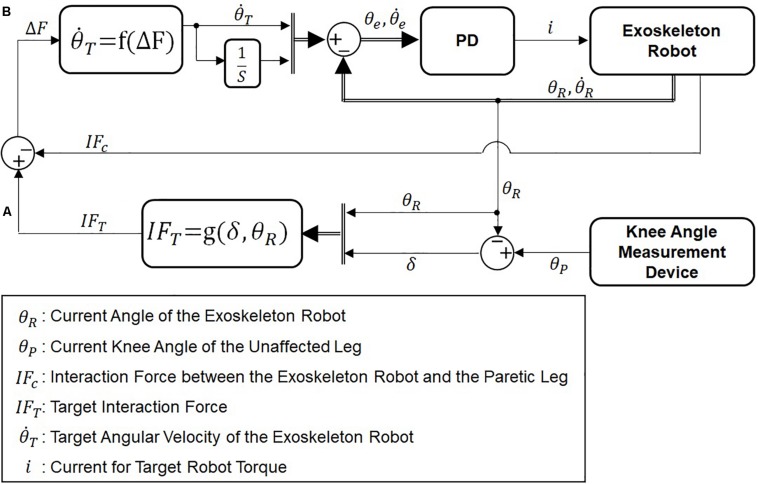
Block diagram of admittance control. **(A)** Calculation of the desired interaction force and **(B)** calculation of the desired angular velocity of ATO.

Figure 4 shows the block diagram of the admittance controller used for the two active modes. First, the target interaction force between the paretic leg and the exoskeleton is calculated based on the difference between the knee joint angles of the unaffected leg and the joint angle of ATO worn on the paretic leg (Figure 4A). The target interaction forces are set differently for the active assist mode and the active resist mode. Detail of setting for the target interaction force in each active mode is described in section “Active Assist Mode” and section “Active Resist Mode.” The difference between the target interaction force and the actual interaction force is calculated. Then, the target joint velocity for PD controller is calculated to reduce the difference between the target and actual interaction force (Figure 4B). The calculation of the target joint velocity has following expressions:

(5) $${\dot{\theta }}_{T}=f\,\left(\Delta \,F\right)=\frac{\Delta \,F}{b}$$

(6) $$\Delta \,F=I\,F_{T}-I\,F_{C}$$

In equation (5), *b* is a constant coefficient between the force difference (△*F*) and the target joint velocity (${\dot{\theta }}_{T}$). In this paper, *b* was used for 30. In equation (6), the force difference (△*F*) is calculated by difference of the target interaction force (*IF*_*T*_) and the actual interaction force (*IF*_*C*_). As the force difference increases, the joint velocity increases in the direction of force difference, which in turn will decrease the interaction force and thus the joint velocity.

### Operation Modes

The developed HRRS can operate in three different levels of difficulty by combining the motion-to-motion interface and the muscle-to-muscle interface. Three operation modes in the developed HRRS is named after the three different control modes in the motion-to-motion interface. Depending on the patient’s stage of motor recovery after stroke (Gowland et al., 1993), the operation mode can be adjusted to provide appropriate amount of support and also encourage maximum involvement by the patient.

#### Passive Mode

In the passive mode, only the motion-to-motion interface is used to provide isokinetic exercise for the paretic leg. Flexion and extension of the paretic leg are commanded by the motion of the unaffected leg through position-based control. The muscle-to-muscle interface is not used in this mode, since the exoskeleton robot produces the movements even without volitional muscle contractions in the paretic leg.

#### Active Assist Mode

In the active assist mode, both the muscle-to-muscle interface and the motion-to-motion interface are used to assist active exercise for the paretic leg through hybrid muscle activation and robotic assistive force.

With the muscle-to-muscle interface, the FES on the paretic leg is modulated by feedback of the EMG signals from both unaffected and paretic legs. The motion-to-motion interface controls the joint displacement and the joint velocity of ATO based on the knee motion of the unaffected leg and the interaction force between the paretic leg and ATO using admittance control.

In this mode, the patient needs to activate one’s RF for knee extension, while muscle activation of the RF is not necessary during knee flexion. The muscle-to-muscle interface compensates for the deficiency in muscle activation of RF on the paretic leg. The motion-to-motion interface assists the motion of the paretic leg based on admittance control as described in section “Motion-to-Motion Interface.”

**FIGURE 5:**
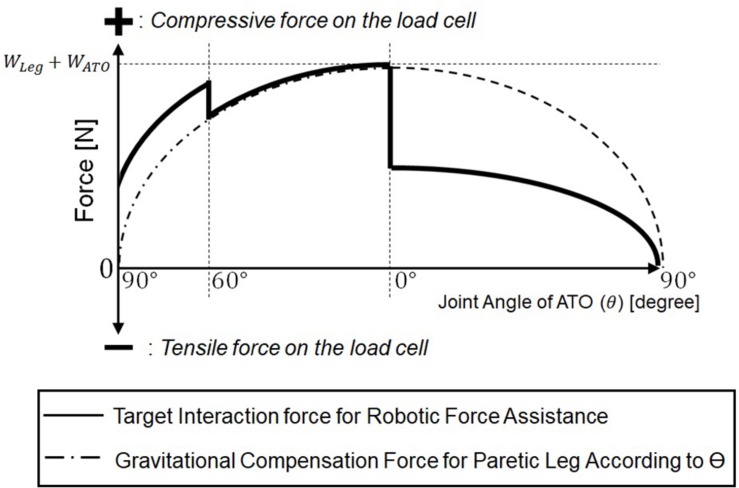
Target interaction force for the robotic force assistance in the active assist mode.

Figure 5 plots the target interaction force for admittance control (solid line). The target interaction force is determined based on the gravity compensation force to counterbalance the weight of the paretic leg and ATO worn on the leg (dashed line). In this figure, the compressive force measured by the load cell is positive, while the tensile force is negative. In this mode, the target interaction force during whole knee movement is set to be compressive to assist the paretic leg in the direction of knee extension. The gravity compensation force, G (θ), related to the joint angle, θ (see Figure 1), has following expression:

(7) $$G\,\left(\theta \right)=\left(W_{L\,e\,g}+W_{A\,T\,O}\right)\,\cos\left(\theta \right)$$

In equation (5), *W*_*Leg*_ and *W*_*ATO*_ are weights of the paretic leg and the ATO. As shown in the Figure 5, the gravity compensation force, G (θ), increases as the joint angle (θ) changes from 90° (Figure 1D) to 0° (Figure 1A), and it decreases as the joint angle changes from 0° (Figure 1A) to 90° (Figure 1D). From 90° (Figure 1D) to 60° (Figure 1C) during knee extension motion, an assistive force larger than the gravity compensation force is applied so that the paretic leg can start the extension motion without any muscle force. From 60° (Figure 1C) to 0° (Figure 1A) during the rest of knee extension motion, an assistive force equal to the gravity compensation force is applied, and FES is applied to the RF on the paretic leg through the muscle-to-muscle interface in case of muscle activation deficiency in the paretic leg. From 0° (Figure 1A) to 90° (Figure 1D) during knee flexion, half of the gravity compensation force is applied to prevent excessive joint velocity during knee flexion, which may cause knee injury.

#### Active Resist Mode

In the active resist mode, both the muscle-to-muscle interface and the motion-to-motion interface are used. With the muscle-to-muscle interface, the FES on the paretic leg is controlled by the difference between the EMG signals from the unaffected leg and the paretic leg.

This mode differs from the active assist mode in that the motion-to-motion interface applies resistive force against the direction of hybrid muscle activation.

In this mode, the patient needs to activate the RF on the paretic leg to overcome the robotic resistive force during both knee extension and knee flexion. This kind of resistance exercise is highly effective for hemiplegia patients in re-gaining muscle strength in their lower limbs (Wist et al., 2016).

The muscle-to-muscle interface compensates for the deficiency in muscle activation of the RF on the paretic leg to overcome the resistive force generated by ATO. During knee extension, the hybrid muscle activation serves for concentric contraction of the RF to overcome the load in the direction of knee flexion. During knee flexion, the hybrid muscle activation serves for eccentric contraction of the RF, while ATO constrains the paretic leg to make knee flexion movement.

In this mode, the target interaction force for the motion-to-motion interface is set to be tensile and constant against the contraction of RF on the paretic leg during knee extension and flexion.

## Experiments and Results

### Experimental Setup

**FIGURE 6:**
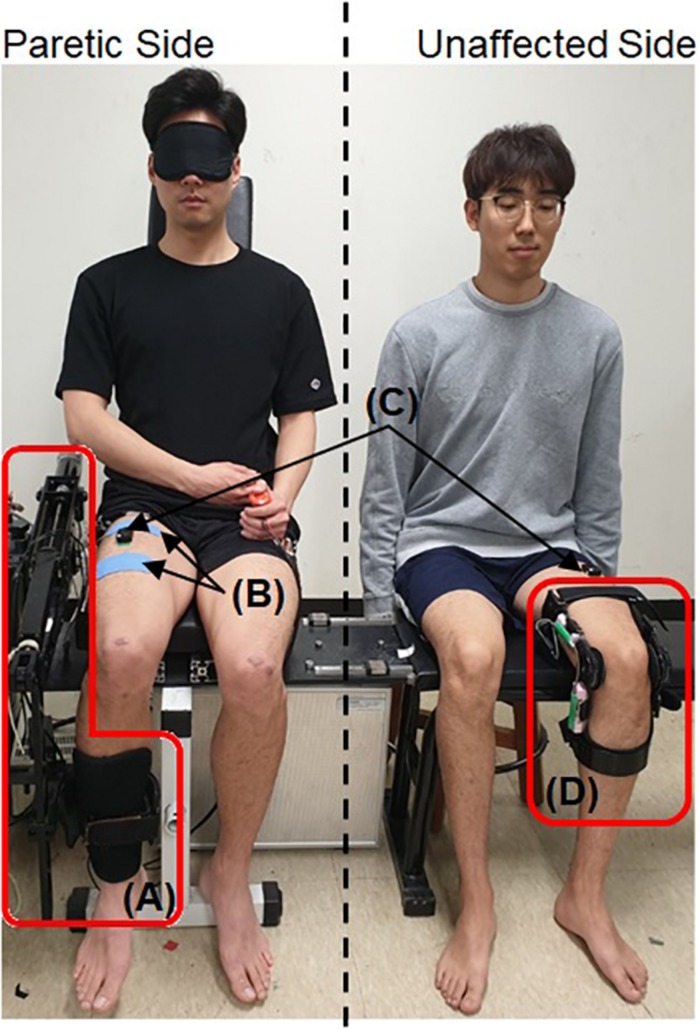
Experimental setup with two healthy subjects. A written informed consent for the publication was obtained from the individuals in this image. **(A)** The exoskeleton robot, ATO, **(B)** the electrodes for the FES, **(C)** sEMG sensors, and **(D)** the self-designed knee angle measurement device.

A total of six healthy subjects aged 25 – 32 participated in this study. The developed system was tested five times, each with two subjects. Each time, one subject (Subject A, B, C, D, or E) served as the paretic side of a hemiplegic patient, while the other subject (Subject G) served as the unaffected side (Figure 6). Each pair of subjects performed the exercise with the three operation modes in same order of (1) Passive Mode, (2) Active Assist Mode, and (3) Active Resist Mode. Each pair of subjects performed each operation mode in different 3 days to minimize learning or fatigue effect. Thus, each operation mode was tested five times by five different pairs of subjects in different days.

The knee angle measurement device and the sEMG sensor were attached to the subject on the unaffected side. The exoskeleton robot, sEMG sensor and FES electrodes were attached to the subject on the paretic side. The subject on the paretic side was blindfolded to prevent any inadvertent muscle activation caused by observing the movement of the subject on the unaffected side. The maximum amplitude of FES applied on the paretic side was limited to 2mA lower than the pain threshold of each human subject. The range of knee movement for the unaffected side which has the same configuration as the joint angle (θ) of ATO (shown in Figure 1) was limited to 5° to 85° for safety.

In the experiment using the Passive Mode, the subject on the paretic side was instructed to neither generate volitional muscle force nor resist the motion of the exoskeleton robot. This is to imitate the state of post-stroke patients in early stages of motor recovery. The subject on the unaffected side was instructed to firstly carry out the knee extension and then to confirm visually that the subject on the paretic side finished the knee extension prior to conducting knee flexion.

In the experiment using the Active Assist Mode, the subject on the paretic side was instructed to generate volitional muscle force only when FES was applied to the RF. The instruction for the subject on the unaffected side was the same as described in the first experiment using the section “Passive Mode.”

In case of the Active Resist Mode, for knee extension, the subject on the paretic side was instructed to generate volitional muscle force only when FES was applied to the RF. For knee flexion, the subject was instructed to volitionally contract the muscle with or without FES application. For the subject on the unaffected side, additional weight was attached to the leg, so that the subject can generate a muscle force larger than that in other operation modes. During knee extension, the subject was required to produce a larger muscle force (concentric contraction) to overcome the extra weight. Also, the subject on the unaffected side was instructed to perform the knee flexion slowly. Due to the extra weight, the subject was required to maintain the muscle activation of the RF during knee flexion (eccentric contraction).

The experiments involving human subjects were approved by the Institutional Review Board at Korea University in Seoul, South Korea (KUIRB-2019-0061-01).

### Comparison of Experimental Results Among the Operation Modes

**FIGURE 7:**
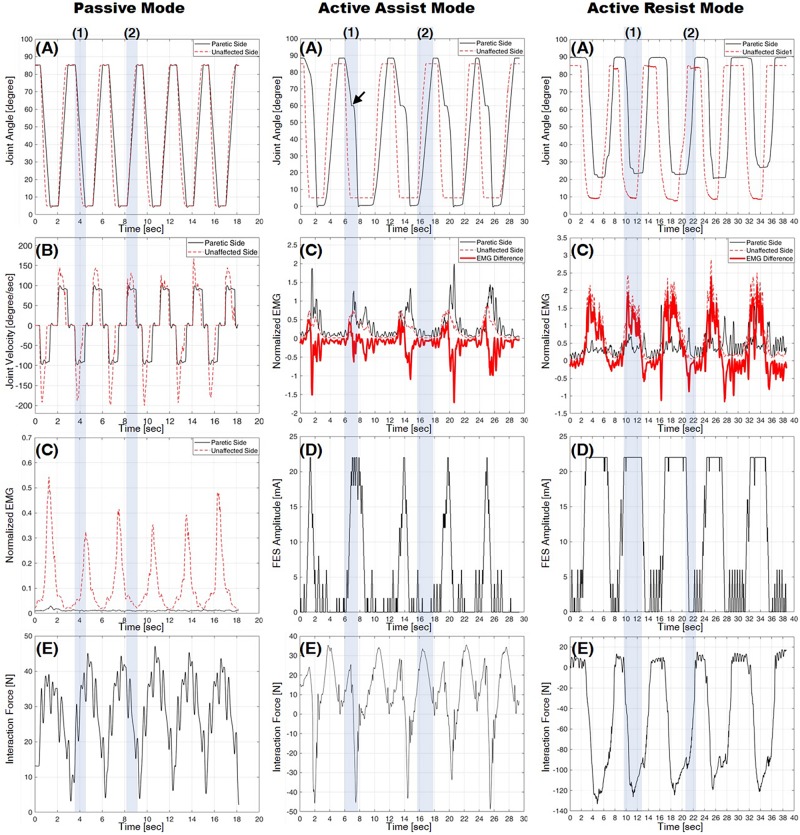
Experimental results from three operation modes: the passive mode in the first column, the active assist mode in the second column, and the active resist mode in the third column. **(A)** Knee joint angle, **(B)** knee joint velocity, **(C)** normalized EMG, **(D)** FES amplitude, and **(E)** interaction force (positive for compression, negative for tension). Shading boxes (1) and (2) indicate the knee extension (from 90° to 0°) and flexion (from 0° to 90°), respectively.

Figure 7 shows the results from one experimental session in three different operation modes described in section “Operation Modes”: the passive mode (shown in the first column), the active assist mode (shown in the second column), and the active resist mode (shown in the third column).

#### Experiments Using Passive Mode

The first column in Figure 7 shows the results from a pair of subjects in the passive mode. Graphs (A) and (B) plot the knee joint angles and velocities on the paretic side (solid line) and on the unaffected side (dashed line), respectively. Graph (C) plots the normalized EMG measurements from the recti femoris on the paretic side (solid line) and the unaffected side (dotted line). The bold line in Graph (C) indicates the EMG difference, which is calculated by subtracting the normalized EMG of the RF on the paretic side from that on the unaffected side. Graph (E) plots the interaction force measured by the load cell. In Graph (A), the knee joint angle of the paretic side follows that of the unaffected side with a small delay. Graph (B) shows that the joint velocity of the paretic side is kept constant owing to the isokinetic control described in section “Passive Mode,” while the joint velocity of the unaffected side is controlled arbitrarily by the subject on the unaffected side.

As can be seen in Graph (C), the normalized EMG of the RF on the paretic side is nearly zero, which indicates that the RF on the paretic side is not volitionally activated unlike the unaffected side.

In Graph (E), the interaction force is kept compressive (positive sign), since the exoskeleton robot counterbalances the weight of the leg while assisting the motion.

The results show that the passive mode of developed HRRS assisted passive exercises at constant joint velocity without any volitional muscle activation from the subject on the paretic side.

#### Experiments Using Active Assist Mode

The second column of Figure 7 shows the results from a pair of subjects in the active assist mode. Graph (A) plots the knee joint angles on the paretic side (solid line) and on the unaffected side (dashed line). Graph (C) plots the normalized EMG measurements from the recti femoris on the paretic side (solid line) and the unaffected side (dotted line). The bold line in graph (C) indicates the EMG difference. Graph (D) plots the amplitude of FES applied to the RF on the paretic side, which is modulated based on the EMG difference, as described in section “Muscle-to-Muscle Interface.” Graph (E) plots the interaction force measured by the load cell. Graph (A) shows that the knee joint angle of the paretic side follows that of the unaffected side with a considerable time delay. The time delay mainly results from the mechanism of the admittance controller, which tracks the target interaction force rather than the target joint angle. During knee extension [shaded box (1)] from 60° (see Figure 1C) to 0° (see Figure 1A), additional muscle force is required to reach full extension (joint angle 0° depicted in Figure 1A), since only the gravitational force is compensated by the exoskeleton robot. As can be seen in the Figure 7 [marked by the arrow in graph (A)], the leg motion stops and waits until additional muscle force is provided by FES.

During knee extension [shaded box (1)], the RF on the unaffected side is activated, and thus the EMG difference increases over the threshold of 0.01, which in turn raises the FES amplitude as shown in graphs (C) and (D). The RF on the paretic side is activated by hybrid muscle activation from both volitional muscle contraction and FES. As the knee flexion [shaded box (2)] starts, the RF on the unaffected side is deactivated, and the EMG difference decreases under the threshold of −0.01, which in turn lowers the FES amplitude. The EMG difference drops to the value between −0.01 and 0.01 since the RF on both sides become deactivated. During knee flexion, the FES amplitude applied to the RF on the paretic side remains nearly zero with no hybrid muscle activation on the paretic side.

During knee extension [shaded box (1)] from 90° (see Figure 1D) to 60° (see Figure 1C), the interaction force is compressive (positive sign) because the leg motion of the paretic side is supported by ATO as shown in graph (E). The EMG [solid line in graph (C)] and the FES amplitude [graph (D)] on the paretic side are nearly zero, which indicates that the hybrid muscle activation is not applied in this range. When the FES is applied at around joint angle 60° (see Figure 1C), the RF on the paretic side becomes activated. This muscle activation accelerates the knee extension motion causing the leg motion to overshoot the motion of ATO. In Graph (E), the tensile interaction force (negative sign) is caused by this overshoot [shaded box (1)]. During knee flexion [shaded box (2)], the interaction force is compressive (positive sign), and the normalized EMG of the RF on the paretic side is close to zero, because the weight of the leg is supported by the exoskeleton robot and muscle activation to counterbalance the weight is unnecessary during flexion.

#### Experiments Using Active Resist Mode

The graphs in the third column of Figure 7 show the results from a pair of subjects in the active resist mode. Graph (A) plots the knee joint angles on the paretic side (solid line) and on the unaffected side (dashed line). Graph (C) plots the normalized EMG measurements from the RF on the paretic side (solid line) and the unaffected side (dotted line). The bold line in graph (C) indicates the EMG difference. Graph (D) plots the amplitude of FES applied to the RF on the paretic side. Graph (E) plots the interaction force measured by the load cell. Graph (A) shows that the knee joint angle of the paretic side follows that of the unaffected side with a considerable time delay, as does in the active assist mode (section “Experiments Using Active Assist Mode”). As can be seen in the Figure 7, the leg on the paretic side did not reach full extension (joint angle 0° depicted in Figure 1A) because even the maximum amount of hybrid muscle activation was not able to overcome the robotic force resistance applied by ATO. As the knee extension starts [shaded box (1)], the RF on the unaffected side produces a large force owing to the extra weight imposed on the unaffected leg. This additional load on the unaffected leg, in turn, increases the FES amplitude on the paretic leg to its maximum value. The hybrid muscle activation from volitional muscle contraction and FES activates the RF on the paretic side during knee extension. Unlike in other operation modes, the muscle on the paretic side is highly activated without FES application during knee flexion [shaded box (2)]. As can be seen in Graphs (C) and (D), the EMG difference shows negative values during knee flexion with nearly zero FES amplitude, which suggests that the RF on the paretic side is more activated than that on the unaffected side to resist the robotic force applied in the direction of knee flexion.

As can be seen in graph (E), the interaction force shows large negative values (tensile force) for both knee extension and knee flexion. The large tensile force is caused by the hybrid muscle activation during the knee extension and the volitional muscle activation during the knee flexion.

### Comparison of Experimental Results Among the Subjects

**FIGURE 8:**
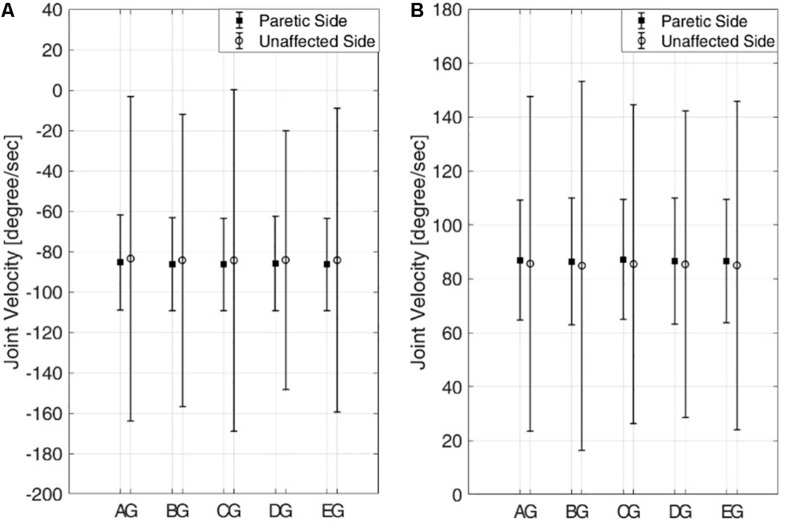
Means and standard deviations of knee joint velocities from five pairs of six subjects in the passive mode. **(A)** Knee extension and **(B)** knee flexion. Six subjects are denoted by A–E,G.

Figure 8 shows the means and standard deviations of the knee joint velocities from the five pairs of six subjects during knee extension (Figure 8A) and knee flexion (Figure 8B) in the passive mode. While the mean velocities from the paretic and unaffected sides are nearly the same, the standard deviations of the joint velocities from the paretic side are much lower than those from the unaffected side for all the pairs of subjects for both knee extension and flexion.

These results indicate that joint motion on the paretic side is kept isokinetic even if the joint motion on the unaffected side, which commands the motion on the paretic side, shows varying joint velocity. Also, the joint velocities of the subjects on the paretic side show no statistically significant differences across the subjects for both extension and flexion (one-way ANOVA with Turkey-Kramer *post hoc* analysis, *p* = 0.71 >0.05 for knee extension and *p* = 0.74 > 0.05 for knee flexion).

**FIGURE 9:**
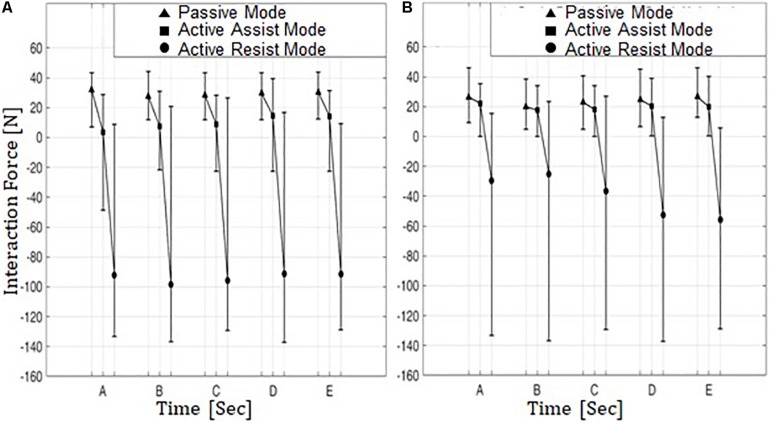
Min-Mean-Max values of interaction force from five subjects on the paretic side in three operation modes. **(A)** Knee extension and **(B)** knee flexion. Five subjects are denoted by A–E.

Figure 9 shows the min-mean-max values of the interaction force for five subjects on the paretic side during knee extension (Figure 9A) and knee flexion (Figure 9B) in the passive mode (triangle), the active assist mode (square), and the active resist mode (circle).

In the passive mode, the interaction forces are compressive (positive sign) for both knee extension and flexion. This indicates that in this operation mode the subjects on the paretic side were passively engaged in knee movement and were relying on robotic motion assistance for conducting the movements.

In the active assist mode, the interaction forces range from compressive (positive sign) to tensile (negative sign) values during knee extension, while the mean interaction forces are slightly compressive. The tensile interaction force can be interpreted as the leg motion outpacing the motion of ATO. This indicates that hybrid muscle contraction plays a considerable role during knee movement with the aid of ATO in the direction of knee extension. During knee flexion, however, the interaction forces are compressive (positive sign), and the mean interaction forces are lower than those in the passive assist mode. This shows that the subjects on the paretic side did not solely rely on ATO during knee flexion, while using the hybrid muscle activation to partially counterbalance the weights of their legs.

In the active resist mode, the interaction forces were mostly in the negative (tensile) range with large negative means for both knee extension and flexion. It appears that in this operation mode the hybrid muscle contraction plays a much larger role in knee movement compared to the other two operation modes. This indicates that the subjects on the paretic side were more actively engaged in the knee movements to overcome the resistive robotic force.

**FIGURE 10:**
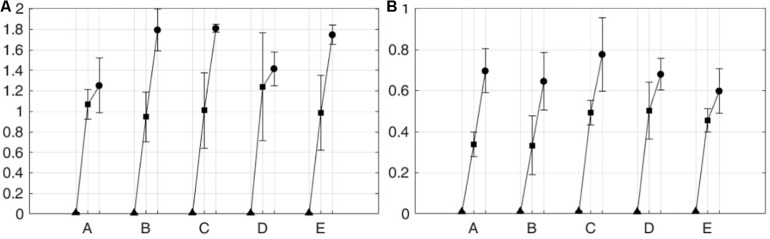
iEMG from five subjects on the paretic side in three operation modes. **(A)** Knee extension and **(B)** knee flexion. Five subjects are denoted by A–E.

Figure 10 shows the means and standard deviations of integrated EMG (iEMG) for five subjects on the paretic side during knee extension (Figure 10A) and knee flexion (Figure 10B) in the passive mode (triangle), the active assist mode (square), and the active resist mode (circle). The iEMG is the time integral of the EMG signal, and it is reported to represent the volitional component of muscle force (Metral and Cassar, 1981). For both knee extension and flexion, iEMG has the maximum mean values in the active resist mode with robotic force resistance (circle) and the minimum mean values in the passive mode with robotic motion assistance (triangle).

Table 1 lists the p-values from t-tests among different operation modes for each subject on the paretic side. As shown in the table, for all the subjects, statically significant differences were observed in iEMG between the passive mode and the two active modes for both knee extension and flexion. Between the two active modes, the iEMG values from all the subjects except for subjects A and D show statistically significant differences for knee extension. For knee flexion, the differences in the iEMG values were statically significant between the two active modes for all the subjects except for subject B.

**TABLE 1:** T-test results among different operation modes.

**Comparing group pairs**	**Subject**	**Knee movement**	
		**Knee extension**	**Knee flexion**
Passive mode V.S. Active assist mode	A	8.9E-05*	0.00027*
	B	0.0045*	0.0073*
	C	0.0039*	6.5E-05*
	D	0.0064*	0.0013*
	E	0.0039*	0.0014*
Passive mode V.S. Active resist mode	A	2.5E-05*	0.00014*
	B	0.0043*	0.0037*
	C	0.00016*	0.00066*
	D	4.3E-05*	4.5E-05*
	E	1.9E-06*	0.0014
Active assist mode V.S. Active resist mode	A	0.29	0.00036*
	B	0.0076*	0.27
	C	5.2E-08*	0.049*
	D	0.51	0.042*
	E	0.0063*	0.04*

*Statistically significant difference with *p* < 0.05.

These results show that volitional muscle activity or active engagement of the subjects on the paretic side can be effectively controlled by the operation modes of the developed HRRS.

## Conclusion

In this study, we developed an exoskeleton-type robotic rehabilitation system for post-stroke patients. For proprioceptive feedback from the unaffected side to the paretic side, the developed robotic system is equipped with two types of interfaces: muscle-to-muscle interface, and motion-to-motion interface.

Unlike the position-based control of conventional bimanual robotic therapies, the developed system is capable of simultaneously stimulating the muscle activities and the joint movements of the paretic limb. Using biofeedback of EMG and functional electric stimulation (FES), the developed rehabilitation system was designed to provide patients with appropriate muscular stimulation considering their stage of motor recovery after stroke. Based on the patient’s condition, the system can be operated in three modes with varying levels of difficulty: the passive mode, the active assist mode and the active resist mode.

The effectiveness of the developed HRRS was tested with five different pairs of healthy human subjects, where one of the two subjects participated as the unaffected side and the other as the paretic side of a hemiplegic patient.

Through repetition of rehabilitation exercises with the developed system, patients can naturally learn the timings at which different muscle groups should be activated to make a joint movement. The methodology developed in this study can be extended to multi-joint rehabilitation systems, such as gait rehabilitation and upper limb rehabilitation.

Further studies are required for clinical application of the developed system. The real-time FES artifact removal technique used in this study needs to be refined to accurately extract volitional components of muscle activity. The efficacy of the developed rehabilitation system should be evaluated with a larger number of post-stroke patients in a clinical setup under supervision of rehabilitation medicine physicians.

## Data Availability Statement

All datasets generated for this study are included in the article/supplementary material.

## Ethics Statement

The studies involving human participants were reviewed and approved by the Institutional Review Board at Korea University in Seoul, South Korea (KUIRB-2019-0061-01). The patients/participants provided their written informed consent to participate in this study. Written informed consent was obtained from the individual(s) for the publication of any potentially identifiable images or data included in this article.

## Author Contributions

JB and SP conceived and designed the interfaces, the muscle-to-muscle interface, the motion-to-motion interface, and merged the interfaces and made three operation modes concerning the rehabilitation stages. S-JK designed and realized the rehabilitation orthosis, ATO, and used for a test bed of the interfaces. JB, SJ, and NP performed the experiments and analyzed the experimental data. Finally, JB wrote the manuscript with the help of SP.

## Conflict of Interest

The authors declare that the research was conducted in the absence of any commercial or financial relationships that could be construed as a potential conflict of interest.
